# Knockout Genes in Bowel Anastomoses: A Systematic Review of Literature Outcomes

**DOI:** 10.3390/jpm14060553

**Published:** 2024-05-23

**Authors:** Georgios Geropoulos, Kyriakos Psarras, Georgios Koimtzis, Massimiliano Fornasiero, Elissavet Anestiadou, Vasileios Geropoulos, Anna Michopoulou, Maria Papaioannou, Kokkona Kouzi-Koliakou, Ioannis Galanis

**Affiliations:** 1 2nd Department of Propaedeutic Surgery, Hippokration Hospital, School of Medicine, Aristotle University of Thessaloniki, 54642 Thessaloniki, Greece; georgios.geropoulos@nhs.net (G.G.); drgxkoimtzis@gmail.com (G.K.); geropoulosv@gmail.com (V.G.); annamichop@yahoo.gr (A.M.); galanis@auth.gr (I.G.); 2 Medical School, University College London, London WC1E6DE, UK; maxfornasiero@googlemail.com; 3 Fourth Surgical Department, School of Medicine, Aristotle University of Thessaloniki, 57010 Thessaloniki, Greece; elissavetxatz@gmail.com; 4 Laboratory of Biological Chemistry, School of Medicine, Aristotle University of Thessaloniki, 54124 Thessaloniki, Greece; mpapaioannou@auth.gr; 5 Laboratory of Histology and Embryology, Medical School, Aristotle University of Thessaloniki, 54124 Thessaloniki, Greece; kkoliako@auth.gr

**Keywords:** knockout genes, anastomosis, rat, surgery, intestinal wound healing

## Abstract

Background: The intestinal wound healing process is a complex event of three overlapping phases: exudative, proliferative, and remodeling. Although some mechanisms have been extensively described, the intestinal healing process is still not fully understood. There are some similarities but also some differences compared to other tissues. The aim of this systematic review was to summarize all studies with knockout (KO) experimental models in bowel anastomoses, underline any recent knowledge, and clarify further the cellular and molecular mechanisms of the intestinal healing process. A systematic review protocol was performed. Materials and methods: Medline, EMBASE, and Scopus were comprehensively searched. Results: a total of eight studies were included. The silenced genes included interleukin-10, the four-and-one-half LIM domain-containing protein 2 (FHL2), cyclooxygenase-2 (COX-2), annexin A1 (ANXA-1), thrombin-activatable fibrinolysis inhibitor (TAFI), and heparin-binding epidermal growth factor (HB-EGF) gene. Surgically, an end-to-end bowel anastomosis was performed in the majority of the studies. Increased inflammatory cell infiltration in the anastomotic site was found in IL-10-, annexin-A1-, and TAFI-deficient mice compared to controls. COX-1 deficiency showed decreased angiogenesis at the anastomotic site. Administration of prostaglandin E2 in COX-2-deficient mice partially improved anastomotic leak rates, while treatment of ANXA1 KO mice with Ac2-26 nanoparticles reduced colitis activity and increased weight recovery following surgery. Conclusions: our findings provide new insights into improving intestinal wound healing by amplifying the aforementioned genes using appropriate gene therapies. Further research is required to clarify further the cellular and micromolecular mechanisms of intestinal healing.

## 1. Introduction

Anastomosis construction is considered one of the most crucial steps in gastrointestinal (GI) surgery. An anastomotic leak is a potentially fatal complication, resulting in higher reoperation rates as well worse prognosis, with increased morbidity and mortality [1,2,3]. Furthermore, apart from the physical and psychological burden, an anastomotic leak represents a significant economic burden for healthcare facilities, mainly due to prolonged hospital stay [4]. Despite progress in GI surgery techniques and materials, GI anastomoses currently present high leakage rates; up to 4% for ileocolic, 18% for colorectal, and 19% for coloanal anastomoses [5]. The problem is exacerbated in patients with inflammatory diseases, sepsis, malnutrition, or in those receiving steroids, immunosuppressives, and neoadjuvant radio-chemotherapy [6].

There are three phases of intestinal wound healing, including inflammation, proliferation, and remodeling, which partly occur simultaneously. The inflammatory phase begins immediately after injury, in which leukocytes infiltrate the submucosa along with accompanying oedema. This process lasts for two weeks, initially with a neutrophil-rich acute response which slowly changes into a chronic macrophage response. Fibroblast and smooth muscle cell proliferation lead to small amounts of collagen formation in order to initially strengthen the anastomosis. Mucosal resurfacing starts early and is completed within one week. Remodeling starts after one week through additional collagen formation and substitution of the already existing collagen with stronger types of collagen. However, the architecture of muscularis mucosa and muscularis propria remains irregular compared to healthy tissue [7].

Intestinal anastomosis healing follows a process quite similar to that encountered in other tissues, with some remarkable differences. The rate of healing in the GI tract is rapid (weeks), whereas in the skin it is more prolonged (months) [8]. The serosa in the GI tract give additional healing strength. The shear stress in the GI tract is increased due to intraluminal bulk transit and peristalsis. GI healing bears a high aerobic and anaerobic bacterial load while skin only has aerobic bacteria. A study from Wahl et al. showed that E. coli species’ predomination in the GI tract can produce toxins, disrupting epithelial integrity and collagen synthesis [9]. Another difference exists in the cellular source of collagen; fibroblasts are responsible for this in the skin, whereas smooth muscle cells are responsible for this in the GI tissues [10]. Furthermore, there are differences in the healing process between different sections of the GI tract. For example, collagen synthesis occurs more rapidly in the ileum compared to the colon [8]. Small bowel anastomosis strength approaches the strength of unwounded tissue about four weeks post injury, while the large bowel needs four months to gain only 75% of its initial strength.

Despite our knowledge of GI healing processes, there are still many molecules and molecule interactions to be identified in order to get a complete idea of their importance and their participation in serious complications, such as improper healing leading to anastomotic leak. A useful tool to explore and analyze the action of certain molecules is the use of knockout mice. Knockout (KO) mice are genetically modified laboratory animals lacking a certain gene and its codified protein. KO mice have been increasingly utilized in recent years to study the immunological and molecular mechanisms involved in anastomotic would healing and interactions therein. This systematic review aims to summarize all significant findings of KO mice experiments in GI wound healing and potentially clarify their relationship to anastomotic leak occurrence.

## 2. Materials and Methods

### 2.1. Study Design and Inclusion/Exclusion Criteria

This systematic review was conducted according to the Preferred Reporting Items for Systematic Reviews and Meta-Analyses (PRISMA) guidelines, in line with the protocol developed and agreed a priori by all authors. Studies investigating the impact of genetic KO mouse or rat models’ effects on bowel anastomosis integrity were deemed eligible for analysis. Control mice or rats among the studies were animals that received no intervention, drinking normal water, or being mentioned in the text as wild-type animals. Exclusion criteria were as follows: (i) articles published in languages other than English, (ii) narrative or systematic reviews and meta-analyses, (iii) case reports, errata, comments, perspectives, letters to the editor, and editorials that did not provide any extractable data, (iv) published abstracts with no available full text, and (v) non-comparative studies (single-arm studies). No publication date, sample size restrictions, or any other search filters were applied.

### 2.2. Search Strategy

Eligible studies were identified by searching through the MEDLINE (via PubMed), Cochrane Library, Embase, and Scopus databases (end-of-search date: 1 October 2023). These searches were conducted by two independent researchers. The search strategies used were the following: (knockout) AND (anastom*). The search algorithm included all of the available synonyms and related terms. Any disagreements were resolved by a third reviewer. The reference lists and all previously published systematic reviews were thoroughly searched for missed studies eligible for inclusion based on the “snowball” methodology [11].

### 2.3. Data Extraction

A standardized, pre-piloted form was used for data tabulation and extraction. Two reviewers extracted the data independently, and any disagreements were identified and resolved by a third reviewer. We extracted the following data from the included studies: study characteristics (first author, year of publication, country, and number of mice in each group), (ii) gene characteristics (KO gene, gene function, underlying condition), (iii) operation characteristics (method of anastomosis, suture material), and (iv) post-operative analysis (hydroxyproline, bursting pressure, histological characteristics).

## 3. Results

### 3.1. Study Characteristics

**Figure 1:**
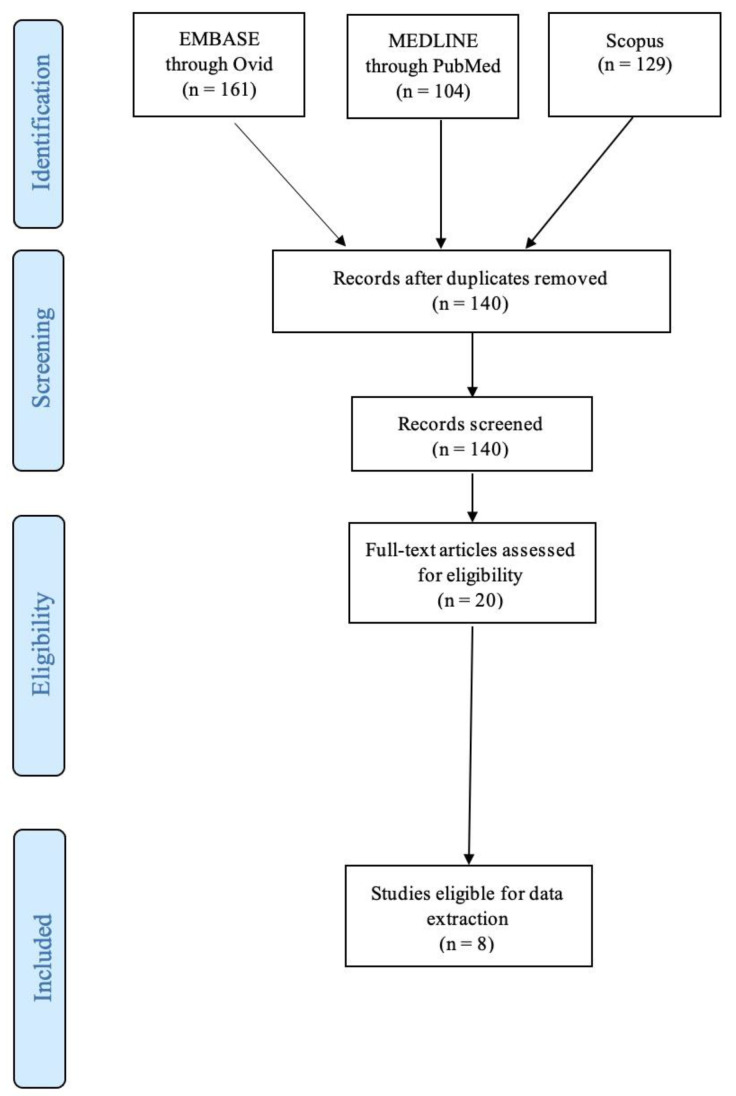
PRISMA flowchart.

A total of one hundred and forty studies were identified and a total of eight studies were included for data extraction, published between 2003 and 2021 [12,13,14,15,16,17,18,19]. Two studies were from Germany [15,16], two studies were from the USA [12,13], another two were from the Netherlands [14,18], one was from Canada [19], and one from China [17]. All studies included KO mouse models. A total of 767 mice were included in the review, averaging 95.9 mice per study (Table 1). A flow diagram is shown in Figure 1.

### 3.2. Experimental Animals Characteristics

Three studies used IL-10 KO mice [12,17,19]. In one of these studies, triptolide was used to reverse the pro-inflammatory effect of IL-10 deficiency [17]. These mice subsequently developed Crohn’s disease as a result of IL-10 deficiency. One study modeled Crohn’s disease by FHL2 deletion, a multifactorial cofactor for gene transcription which negatively regulates mitogen-activated protein kinase (MAPK) signaling [16]. Another study used cyclooxygenase-2 (COX-2) KO mice mimicking a non-steroidal anti-inflammatory drug (NSAID) effect. The result was immune modulation, myoblast proliferation, and reduced vascular-endothelial-growth-factor- (VEGF-) induced angiogenesis [14]. One study used annexin A1 (Anxa1) gene KO mice to induce inflammatory colitis by leukocyte activation and transmigration [15]. Another study used thrombin-activatable fibrinolysis inhibitor (TAFI) KO mice, compared to wild type (WT) mouse models, for anastomotic studies [18]. One study used heparin-binding epidermal growth factor (HB-EGF) KO mice for their experiments, again compared to WT mice used as control [13] (Table 1).

### 3.3. Operative Characteristics

The majority of studies used interrupted sutures to form an end-to-end anastomosis [12,13,14,15,16,17]. One study used continuous sutures for their end-to-end anastomosis [18] and another study formed a side-to-side anastomosis with interrupted sutures [19]. Three studies used 8-0 prolene for the anastomosis [14,18,19]. Two used 9-0 nylon [12,17], and one used 4-0 nylon [13]. Two studies used vicryl sutures [15,16] (Table 2).

### 3.4. Histological Characteristics

All studies assessed the histological characteristics of the bowel anastomosis in gene KO mice. In IL-10 KO mice, a study found no significant difference in collagen deposition between IL-10 KO mice and control [19], while another study found significantly increased collagen mRNA in IL-10 KO mice compared to WT mice. This additional collagen mRNA translated into increased collagen deposition and fibrosis at the anastomosis [12]. The microbiome is also likely to play a role, with reduced fibrosis in germ-free IL-10 KO mice compared to conventional IL-10 KO mice [12], although the exact mechanisms have not been identified. Wu et al. [17] found epithelial cell hyperplasia, mucin depletion, and crypt abscess formation in IL-10 deficient mice at the anastomosis site and more extensively throughout the bowel.

FHL2 KO mice demonstrated reduced submucosal collagen type I and III deposition around the injury site, with no difference in collagen in areas of unaffected bowel compared to WT mice acting as controls [16]. Inhibition of COX-2 correlated with reduced vascularization around the anastomosis site compared to controls [14]. Annexin A1 KO mice demonstrated a reduced histological healing score (Scored 0–4 for inflammatory cells, angiogenesis, collagen synthesis, fibroblast ingrowth, and overall healing quality, adapted from Phillips et al. [20]) compared to control, which was improved with the addition of Ac2-26 nanoparticles [15]. TAFI KO mice showed no differences in granulation tissue deposition between the KO group and WT [18]. HB-EGF KO mice showed reduced healing scores compared to WT. Transgenic (TG) HB-EGF mice, engineered to overexpress HB-EGF resulting in additional HB-EGF produced, showed increased collagen deposition and mature vascularization around the anastomosis site compared to WT mice [13]. Seven studies assessed inflammation differences around the anastomosis site [12,13,14,15,17,18,19]. IL-10 KO mouse models showed increased lymphocyte infiltration when compared to WT mice that faced either surgical intervention or a non-operative study arm [12,17,19]. This was reversed with administration of triptolide [17]. Increased inflammation (defined as increased numbers of morphonuclear leukocytes, lymphocytes, and macrophages on histological analysis) was found in HB-EGF KO, compared to WT, which demonstrated increased inflammation compared to TG HB-EGD mice [13]. Increased inflammation and matrix metalloproteinase (MMP) expression was found in Anxa1 KO mice compared to WT, with reduced inflammation upon addition of Ac2-26 nanoparticles [15]. However, no anastomotic area inflammatory differences were noted between COX-2 KO mice and WT mice [14]. Interestingly, Te Velde et al. [18] only found increased inflammation around the anastomotic site in TAFI KO mice who experienced anastomotic leak, but not in other TAFI KO mice (Table 2).

### 3.5. Bursting Pressure

Three studies assessed bursting pressure. Kirfel et al. [16] demonstrated a reduced bursting pressure in FHL2 KO mice compared to control at days 2, 5, and 14, postoperatively. TAFI KO mice also had a reduced bursting pressure despite no granulation tissue differences when compared to WT mice [18]. HB-EDF KO mice had reduced bursting pressure compared to WT mice. TG HB-EGF mice had increased bursting pressure when compared to WT, again correlating with collagen deposition noted on histological analysis [13] (Table 2).

## 4. Discussion

Bowel resection and anastomosis represents a significant component of GI disease surgical management. Anastomotic leak represents a severe post-operative complication, with varying incidence up to 36% [21]. The clinical presentation of leaks varies significantly, from low-grade fever and overall delayed recovery of the patient to severe peritonitis and sepsis [22]. This results in longer inpatient stays, increased mortality, and higher healthcare expenses [23,24,25]. Therefore, it is of utmost importance to elucidate intestinal wound healing pathways in order to find ways to improve healing and reduce the incidence of anastomotic leaks. This systematic review identified several studies investigating the lack of the following molecules: IL-10, cyclooxygenase-2, annexin A1, TAFI, and heparin-binding epidermal growth factor genes. Increased inflammatory cell infiltration in the anastomotic site was found in IL-10-, annexin-A1-, and TAFI-deficient mice compared to controls. COX-1 deficiency showed decreased angiogenesis at the anastomotic site. Our findings provide new ways to improve intestinal wound healing through amplifying the aforementioned genes with appropriate gene therapies. Genetic modification of mouse models has translated into different results of clinical parameters. Various agents have been shown to be useful in reducing anastomotic leaks and their relative complications. For example, Radulescu et al. [13] generated mouse models over-expressing HB-EGF. This increased bursting pressure and collagen deposition. In a different included study conducted by Reischl et al., Ac2-26 nanoparticles added to ANXA1 KO mice improved histological healing [15]. Addition of prostaglandin E2 in COX-2-deficient mice partially improved anastomotic leak rates, closer to those of WT mice [14]. HB-EGF KO mice models led to higher mortality and complication rates (including bleeding, anastomotic dehiscence, abscess formation, and obstruction) [13]. Treatment of ANXA1 KO mice with Ac2-26 nanoparticles reduced colitis activity and increased weight recovery following surgery [15]. Use of diclofenac to inhibit COX-2 resulted in significantly increased rates of anastomotic leak compared to control [14]. TAFI KO mice had significantly increased weight loss, adhesions, bleeding, and mortality compared to WT mice [18].

### 4.1. Interleukin 10, miR-155/SHIP-1 Pathway and Wound Healing

IL-10, as a regulatory cytokine of inflammation, has been the subject of extensive study in the context of anastomotic healing. IL-10 is primarily produced from macrophages residing in the lamina propria of the bowel and regulatory T-cells in order to reduce the immune response to proinflammatory molecules on cell membranes of local bacteria [26]. In addition, epithelial cells and Th1 cells triggered by lipoproteins of commensal bacteria are capable of producing IL-10 in order to maintain bowel homeostasis [27,28]. Incubation of IL-10 KO mice’s intestinal epithelial cells with recombinant IL-10 enhanced wound repair in vitro because IL-10 binding reversed the KO effect [29]. In vivo, macrophage-derived IL-10 was crucial for small intestine epithelial healing during the acute phase of injury, ultimately driving re-epithelialization in the small intestine [30]. The exact mechanism through which IL-10 promotes bowel wound healing remains unclear. Following biopsy-induced injury, IL-10 activates cyclic adenosine monophosphate (c-AMP) Response-Element-Binding Protein (CREB) signaling and its downstream target WNT1-inducible-signaling pathway protein 1 (WISP-1). This, in turn, promotes β-catenin signaling to induce epithelial proliferation and wound closure [31]. It is therefore possible that IL-10 works directly on epithelial cells. An alternative mechanism suggests that IL-10 works primarily on immune cells, resulting indirectly in wound healing. IL-10 binding to CD11+ myeloid cells regulates T cell activation and proliferation of intestinal crypt cells [12,32]. The broad functions of IL-10 have generated significant interest in it as a therapeutic target for multiple disease states. Significant research involving manipulation of IL-10 has been undertaken in the context of inflammatory bowel disease (IBD) [25]. IL-10-deficient mice develop colitis [33], which is managed successfully following administration of IL-10 [34,35]. In 1993, Kühn et al. [36] showed that interleukin-10-deficient (IL-10−/−) mice presented a Th1-mediated chronic colitis similar to human IBD. However, administration of recombinant IL-10 yielded only a minor clinical improvement in the treatment of IBD in humans [37], likely due to the low concentrations achieved in the gut following systemic administration. Lactococcus lactis bacteria, modified to deliver IL-10 to the gut, improved clinical disease scores of a small number of Crohn’s disease patients [38]. The effects of IL-10 have also been studied in dermal wound healing. Fetal skin has a tendency to regenerate, whereas postnatal skin heals with scar formation. Fetal skin has elevated levels of IL-10, promoting high molecular weight hyaluronan formation, which reduces inflammation and fibrosis [39,40]. IL-10 administration in IL-10-deficient mice resulted in reduced inflammatory cell numbers and restoration of normal skin architecture, with reduced scar size. This resulted in accelerated dermal healing and increased tissue strength [41]. Regarding IBD, it has been demonstrated that triptolide ameliorates the severity of IL-10-deficient mouse colitis via inhibition of TLRs (toll-like receptors)/NF-κB and IL-6/STAT3 signaling pathways, and down-regulation of IL-17 [42,43]. Triptolide is a bioactive compound produced by the plant Tripterygium wilfordii Hook F (TwHF) and has been widely used in clinical practice due to its anti-inflammatory and immunosuppressive properties [44]. Its mechanisms of action include the following: (1) inhibition of expression of RPB1 (the RNA polymerase II main subunit), (2) inhibition of xeroderma pigmentosum group B (XPB) protein, which is a subunit of transcription factor II-H, and (3) inhibition of induction of miR-155 in LPS-stimulated macrophages [17]. In particular, miR-155 targets SHIP-1 (Src-homology-2-containing inositol phosphatase-1), which is an inhibitor of many inflammatory processes and regulates the balance of T cell subsets [17,42,43].

### 4.2. Cyclooxygenase (COX) and Wound Healing

COX is an essential enzyme in the synthesis of prostaglandins, occurring in two isoforms. COX1 is constitutively expressed, whereas COX2 is expressed in response to injury. In dermal injury, COX-2 expression peaks 3 days later, when the anastomosis is thinnest and at increased risk of rupture [45]. The quantity of COX-2 produced also correlates with the magnitude of the inflammatory response [46]. Hypoxia, partly due to connective tissue deposition during wound healing, stimulates angiogenesis through HIF-1α induction of VEGF secretion. VEGF is also responsible for fibroblast differentiation and tissue remodeling. The COX–VEGF axis plays an important role in wound healing, since pharmacologically induced downregulation of COX-2 correlates with significantly increased VEGF levels. A study of full-thickness skin incisions in mice demonstrated increased angiogenesis and collagen deposition on histology, which correlated with a significant increase in wound contraction and reduced wound area macroscopically [47]. These findings are in contrast to those of another paper included in our analysis, indicating that COX-2 KO mice demonstrate reduced VEGF-induced angiogenesis at the anastomosis [14]. This could be due to an alternative mechanism for VEGF production, or a difference in healing between dermal and bowel injury. However, COX-2 and the resulting production of prostaglandin E2 have been shown to accelerate healing of gastric ulcers, further demonstrating the importance of this pathway in gastrointestinal mucosal repair [48]. Prostaglandin (PG) biosynthesis is mediated by the synergic action of two enzymes: phospholipase, responsible for the metabolism of arachidonic acid (AA) from cell membrane phospholipids, and COX. Prostaglandin E2 (PGE2) is the most abundant and important COX-2 product regarding intestinal wound healing [49]. Under normal conditions, the PGE2 signaling pathway can potentially trigger stem cell differentiation towards enterocytes [50]. After an injury, epithelial restitution is a crucial process that is performed in the intestine by a special cell population with transient repair features named wound-associated epithelial cells (WAE) and, in combination with non-canonical Wnt signaling activation, this ensures controlled wound repair [51]. Epithelial restitution is a protective mechanism including the migration of epithelial cells from intact wound margin into the injured basal lamina to reseal the intestinal barrier. Miyoshi et al. showed that PGE2 signaling through its receptor promotes WAE differentiation of intestinal epithelial stem cells in an in vitro model. In vivo studies in mice with depletion of the PGE2 receptor led to insufficient intestinal wound repair due to impaired production of WAE cells, and they exhibited impaired wound repair [52]. In addition, it has been found that increased COX-2 expression is noted in macrophages and myofibroblasts after exposure to proinflammatory signals, triggering intestinal tissue proliferation [14]. Numerous studies have shown the regenerative role of PGE2, a product of the COX-2 gene, for intestinal wall repair under a wide range of pathological circumstances, such as dextran-sulfate-sodium- (DSS-) induced inflammation and ischemia-reperfusion injury [53,54]. In addition, PGE2 and COX-2, through activation of the PGE2 receptor and following upregulation of VEGF expression, stimulate neoangiogenesis but also promote the proliferation and migration of epithelial intestinal cells [55]. Finally, PGE2 regulates myofibroblast function, which is responsible for producing collagen, the main structural component of the extracellular matrix [56]. In terms of clinical application, there is conflicting evidence regarding whether COX-2 blockage is associated with impaiastomotic leak development. Two recent meta-analyses involving patients undergoing operations for colorectal cancer have concluded in undetermined relationships between postoperative NSAID administration and anastomotic leakage [57,58]. On the other hand, a subgroup analysis by Jamjittrong et al. focused on colorectal anastomoses revealed a significantly increased anastomotic leakage rate in patients who were administered NSAIDs perioperatively [57]. In human genome studies, the PTGS2 −765G > C polymorphism lies in the promoter region of the gene that encodes COX-2 and leads to lower levels of COX-2 [59]. Reisinger and Makar suggested that the homozygous state for PTGS2-765G > C polymorphism, which is encountered with a prevalence of 3% in humans, led to decreased COX-2 expression and increased anastomotic leakage rates [14,60].

### 4.3. Anxa1 and Wound Healing

Annexin A1 (AnxA1), also named lipocortin-1, is a calcium-dependent phospholipid-binding protein from the family of SPMs. Clinical studies have shown that higher AnxA1 expression is associated with milder symptomatology and severity in both Crohn’s disease (CD) and ulcerative colitis (UC) [61]. A 26-amino acid ANXA1 N-terminal peptide Ac2-26, a part of the full-length molecule, is the active part of ANXA1 [62]. AnxA1’s anti-inflammatory role is based on inhibition of neutrophil accumulation, transendothelial migration, and activation, as well as promotion of neutrophil apoptosis after binding to the formyl peptide receptor 2 (FPR2/ALX) and formyl peptide receptor 1 (FPR1) [15]. In addition, AnxA1 induces monocyte chemotaxis and removal of apoptotic leukocytes by macrophages. It promotes keratinocytes’ motility and differentiation, which is important for wound re-epithelization [63]. Finally, AnxA1 controls macrophage cell reprogramming towards reduced production of proinflammatory molecules and increased production of anti-inflammatory agents [64]. Annexin 2 is a calcium-dependent phospholipid-binding protein associated with cytoskeleton coordination [65]. Through annexin 2 [66], epithelial cell migration re-establishes the epithelial barrier and reseals the mucosal defect of the bowel following surgery. Our review included a study [15] investigating the role of annexin A1 on the resolution of inflammation following surgery for colitis. Epithelial injury of the bowel brings into contact the immune cells of the intestinal wall and the lumen’s bacteria, resulting in an inflammatory response. Anxa1 is a component of the extracellular vesicles of these immune cells [67]. When released into the extracellular space, it modifies the inflammatory response of phagocytes and epithelial cells through binding to formyl peptide receptors [68]. Our included study [15] found an increased inflammatory score and increased MMP in Anxa1 KO mice, which is consistent with the process described above. Therefore, Anxa1 deficiency reduces epithelial integrity by exposing inflammatory cells to the gut microbiome. Clinically, increased Anxa1 expression in mouse models of CD correlated with histological recovery of the gut mucosa [69]. Further evidence suggests that Anxa1 mediates the positive response of infliximab in CD patients [70].

### 4.4. FHL2 and Wound Healing

FHL2 belongs to the LIM protein superfamily and exhibits a key role in a wide range of cell functions, including regulation of cell proliferation, gene expression, survival, control of cell architecture, cell adhesion, apoptosis, cell motility, and signal transduction [71]. FHL2 has a distinctive signaling role for mesenchymal cells, controlling their migration, contraction, and differentiation into myofibroblasts through expression of α-smooth muscle actin [16]. During the inflammation phase, FHL2 contributes to pathogen clearance, synthesis, and release of proinflammatory cytokines and immune cell infiltration. During the migration/proliferation phase it has a key role in the function of myofibroblasts, in their differentiation from fibroblasts or from epithelial cells via epithelial mesenchymal transition, and in suppression of MMPs, leading to sufficient contraction and formation of granulation tissue. Finally, during the remodeling phase of the wound healing, FHL2 participates actively in ECM synthesis and remodeling [72]. The literature contains many reports regarding the role of FHL2 in cardiovascular disorders and carcinogenesis [73,74]. However, emerging evidence indicates its role in all phases of wound healing and inflammation. Although under normal conditions FHL2 expression is very low in most tissues, it is upregulated in the skin during wound repair, especially in phases of migration and proliferation. FHL2 suppression is associated with healing failure or prolonged wound healing [72]. In terms of anastomosis healing, Kirfel et al. [16] explored the healing of an end-to-end small bowel anastomosis in FHL2-deficient mice compared to WT mice. They found that FHL2 KO mice presented a statistically significant increase in anastomotic failure rate and lower anastomotic bursting pressure. Histopathologically, insufficient wound healing was noted in anastomotic specimens derived from KO mice, characterized by decreased mucosal and muscular continuity and reduced granulation tissue formation at the fifth and fourteenth postoperative day. On the fifth postoperative day, WT mice presented a dense pattern of organized collagen formation, mainly in the submucosal layer of injured tissue, while submucosa in the FHL2 KO mice presented significantly thinner collagen I/collagen III networks. This was due to downregulation of collagen III mRNA expression in FHL2 KO mice at the anastomotic site. Depletion of FHL2 did not lead to difference in local MMP expression [16].

### 4.5. HB-EGF and Wound Healing

HB-EGF belongs to the epidermal growth factor (EGF) family. It binds to heparin, as well as to human epidermal growth factor receptors ErbB-1 and ErbB-4, and triggers cellular proliferation, migration, adhesion, and differentiation [75]. Especially for intestinal tissue, it has been shown that HB-EGF exerts a protective effect on intestinal epithelial cells, intestinal stem cells, immunocytes, vascular endothelial cells, pericytes, and intestinal neuronal cells after injury by preserving their cytoskeletal structure and proliferative potential [76,77]. Furthermore, its role in intestinal injury has been delineated in experimental models of necrotizing enterocolitis [78], ischemia/reperfusion injury [79], and resuscitation after hemorrhagic shock [80]. It has been demonstrated that HB-EGF repairs the intestinal epithelium via a phosphatidylinositol 3-kinase (PI3K) and extracellular signal-regulated kinase (ERK)1/2 pathways [81]. Angiogenesis and capillary formation are the basic mechanisms of action of the HB-EGF molecule, which has been shown both in vitro and in vivo [82]. To investigate the role of HB-EGF in intestinal anastomotic healing, Radulescu et al. [13] studied outcomes after division and re-anastomosis of the terminal ileum in HB-EGF KO mice and HB-EGF transgenic (TG) mice. In addition, an extra group of WT mice receiving per os HB-EGF (800 µg/kg) was also included in the experiment [13]. HB-EGF KO mice showed significantly reduced anastomotic bursting pressure and impaired healing scores on the sixth postoperative day. Moreover, higher mortality and greater complication rates postoperatively were reported in HB-EGF KO mice compared to normal controls. Histopathologically, lower collagen levels, severe inflammation in the submucosal space around the anastomosis, and reduced angiogenesis were noted in KO mice. In contrast, HB-EGF TG mice presented increased anastomotic bursting pressure and higher healing scores on the sixth postoperative day, similar mortality, and lower complication rates postoperatively. Histological examination revealed increased angiogenesis and collagen production, minimal signs of inflammation, improved epithelialization, and significant submucosal healing. Per os administration of HB-EGF showed increased anastomotic bursting pressure and significantly lower complication rates. These findings are supportive of the possible cytoprotective role of HB-EGF for intestinal epithelial cells [83].

## 5. Conclusions

In conclusion, we have identified numerous genes which play crucial roles in anastomotic healing as evidenced by mouse models. IL-10 plays a role in regulating the immune response to prevent excessive inflammation. FHL-2 gene products and HB-EGF improve the structural integrity of the anastomosis, promoting collagen deposition and reducing the bursting pressure. COX-2 gene products are important for signaling VEGF production, contributing to vascularization of the anastomosis.

## Figures and Tables

**Table 1:** Summary of the study details and genetic knockout characteristics. ANXA1 = annexin A1, COX-2 = cyclooxygenase-2, HB-EGF = heparin-binding epidermal growth factor, IL-10 = interleukin-10, KO = knockout, MAPK = mitogen-activated protein kinase, NA = not applicable, NSAID = non-steroidal anti-inflammatory drug, SHIP1 = Src homology-2 domain-containing inositol 5-phosphatase 1, TAFI = thrombin-activatable fibrinolysis inhibitor, TG = transgenic, VEGF = vascular endothelial growth factor, WT = wild type (act as control for the genetically altered mice).

Study (Year)	Country	Knockout Gene	Gene Function	Underlying Condition	Groups (Number of Participants, *n*)
Borowiec et al. (2012) [19]	Canada	IL-10 129/SvEv background (the strain of mice used for experimentation)	IL-10 production	Crohn’s disease	Control WT (no intervention) (*n* = 18) Control IL-10 null (no intervention) (*n* = 18) Ileocolonic anastomosis WT (*n* = 18) Ileocolonic anastomosis IL-10 null (*n* = 18) Sham surgery WT (*n* = 18) Sham surgery IL-10 null (*n* = 18)
Wu et al. (2013) [17]	China	miR-155/SHIP-1 pathway, responsible for epigenetic modification, altering transcription rates and cell turnover	Th1/Th2 regulator Increased miR-155 promotes differentiation into CD4+ cells and inhibits CD8+ differentiation	Crohn’s disease	IL-10 KO control (no intervention) (*n* = 6) IL-10 KO ileocecal anastomosis + intraperitoneal saline injection (*n* = 6) IL-10 KO ileocecal anastomosis + intraperitoneal triptolide injection (*n* = 6) WT control (no intervention) (*n* = 6)
Kirfel et al. (2008) [16]	Germany	FHL2	Multifactorial cofactor for gene transcription, negatively regulates MAPK signaling	Crohn’s disease	WT (*n* = 68) FHL2-deficient mice (*n* = 70)
Rigby et al. (2009) [12]	USA	IL-10 129/SvEv background	IL-10 production	Crohn’s disease	WT ileo-cecal resection (*n* = 10) WT sham (*n* = 8) WT no surgery (*n* = 3) IL-10 null ileo-cecal resection (*n* = 11) IL-10 null sham (*n* = 6) IL-10 null no surgery (*n* = 6)
Reisinger et al. (2017) [14]	The Netherlands	COX-2	Immune modulation, myofibroblast proliferation, VEGF-induced angiogenesis through prostaglandin production	NA—mimic NSAID use	WT (*n* = 32) COX-2 + /− (*n* = 17) COX-2 KO (*n* = 25)
Reischl et al. (2021) [15]	Germany	ANXA1	Annexin A1 generation—anti-inflammatory protein inhibiting leukocyte activation and transmigration	Colitis	WT-induced colitis (*n* = 36) Anxa1-KO-induced colitis (*n* = 36) Colitis + anastomosis (*n* = 56) Control (normal drinking water without DSS) + anastomosis (*n* = 36) Colitis + anastomosis + Ac2-26-nanoparticles (*n* = 24) Colitis + anastomosis + sham Ac2-26-nanoparticles (*n* = 24)
Te Velde et al. (2003) [18]	The Netherlands	TAFI	Downregulates fibrinolysis	NA	WT mice (*n* = 14) TAFI knockout mice (*n* = 14)
Radulescu et al. (2011) [13]	USA	HB-EGF	Mitogen, chemoattractant for smooth muscle cells and fibroblasts	NA	HB-EGF KO (*n* = 42) HB-EGF +/+ WT (*n* = 33) HB-EGF TG mice (*n* = 26) WT (*n* = 27) WT mice with enteral HB-EGF added (*n* = 21)

**Table 2:** Summary of operative details, histological and inflammatory analysis, and bursting pressure. ANXA1 = annexin A1, COX-2 = cyclooxygenase-2, HB-EGF = heparin-binding epidermal growth factor, IL-10 = interleukin-10, KO = knockout, MMP = matrix metalloproteinase, NA = not assessed, POD = postoperative day, TAFI = thrombin-activatable fibrinolysis inhibitor, TG = transgenic, VEGF = vascular endothelial growth factor, WT = wild type.

Study (Year)	Method of Anastomosis	Suture Material	Histology	Bursting Pressure	Inflammatory Effect
Borowiec et al. (2012) [19]	Interrupted sutures, side-to-side anastomosis	8-0 Prolene	No collagen difference between WT and IL-10 null mice	NA	Increased lymphocyte infiltration in IL-10 KO
Wu et al. (2013) [17]	Interrupted sutures, end-to-end anastomosis	9-0 nylon	Epithelial cell hyperplasia, mucin depletion, and crypt abscess formation in anastomosis group, reversed by administration of triptolide	NA	Increased lymphocytic infiltration in anastomosis group compared to control, reversed by administration of triptolide
Kirfel et al. (2008) [16]	Interrupted sutures, end-to-end anastomosis	8-0 polyglactin	Reduced submucosal collagen type I and III deposition in FHL-2-deficient mice around injury site, no difference in unaffected bowel compared to control	Lower bursting pressure in FHL-2-deficient mice at days 2, 5, and 14 postoperatively	NA
Rigby et al. (2009) [12]	Interrupted sutures, end-to-end anastomosis	9-0 nylon	Increased collagen mRNA in small intestine of IL-10 null mice compared to WT Reduced fibrosis in germ-free IL-10 null mice compared to conventional IL-10 null mice	NA	Inflammation in IL-10 null mice after ileo-cecal resection in the small intestine and anastomosis No difference between WT mice of different treatment groups
Reisinger et al. (2017) [14]	Interrupted sutures, end-to-end anastomosis	8-0 Prolene	Significantly reduced vascularization, reduced VEGF levels in COX-2 KO compared to WT	NA	No inflammatory differences observed between WT and COX-2 KO
Reischl et al. (2021) [15]	Interrupted sutures, end-to-end anastomosis	9-0 Vicryl	Increased MMP expression on POD1 in ANXA1 KO compared to WT mice Ac2-26 nanoparticles improved histological healing scores compared to control	Healthy mice	Higher inflammation score in Anxa1 KO mice compared with WT Ac2-26 nanoparticles reduced colitis activity and suppressed pro-inflammatory cytokine expression
Te Velde et al. (2003) [18]	Continuous sutures, end-to-end anastomosis	8-0 Prolene	POD7: no gross differences in granulation tissue between KO and WT mice	Significantly lower in TAFI KO compared to WT Burst on anastomosis line in 4/14 TAFI KO mice, but normal intestinal tissue in all WT mice	Increased inflammation only in 4/14 TAFI KO mice following anastomotic leak
Radulescu et al. (2011) [13]	Interrupted sutures, end-to-end anastomosis	4-0 Nylon	KO mice demonstrated worse healing scores compared to WT, which were worse than HB-EGF TG Increased collagen deposition and increased mature vascularization of anastomosis in TG mice compared to KO mice	HB-EGF KO < WT < HB-EGF TG	KO mice demonstrated increased inflammation compared to WT, which was increased compared to HB-EGF TG

## Data Availability

No new data were created. Data taken from previously published studies have been cited in this article.
